# European Psychiatric Association policy paper on ethical aspects in communication with patients and their families

**DOI:** 10.1192/j.eurpsy.2020.33

**Published:** 2020-04-01

**Authors:** Bernardo Carpiniello, Danuta Wasserman

**Affiliations:** 1 Department of Medical Science and Public Health, Psychiatric Unit, University Hospital Cagliari, Cagliari, Italy; 2 National Centre for Suicide Research and Prevention of Mental Ill-Health (NASP) LIME, Karolinska Institutet-CHIS, Stockholm, Sweden

**Keywords:** Communication, diagnosis, ethical issues, mental disorders, treatments

## Abstract

**Background.:**

Establishing a valid communication is not only a basic clinical need to be met but also a relevant ethical commitment.

**Methods.:**

On the basis of the relevant literature, ethical issues arising from specific, important situations in clinical practice were identified.

**Results.:**

The main ethical problems regarding communication about the disorder, both in general and in relation to prodromal stages, were described and discussed together with those regarding communication about voluntary and involuntary treatments, “dual roles” enacted in clinical practice, genetic counseling, and end-of-life conditions; on the basis of what emerged, ethically driven indications and suggestions were provided.

**Conclusions.:**

Several situations put the psychiatrist in front of relevant dilemmas and doubts which are no easy to face with; an ethically driven approach based upon the principle of the best interest of patients may support clinicians in their decisions.

## Introduction

The quality of psychiatrist-patient relationships plays a central role in engaging subjects in treatment and obtaining positive outcomes [1,2]. In the context of therapeutic relationships, good communication is fundamental basis for establishing an effective partnership with clients, indeed, efficacy in communication seems to be crucial in developing positive relationships with patients based on openness and trust [3]. In other words, communication seen either as an interpersonal process of exchange of information on a cognitive and affective level [4] or as a simultaneous two-way interpersonal experience [5], plays a fundamental role in establishing a valid relationship with patients, and possibly with their families and caregivers. Communication per se may be considered a therapeutic tool, particularly as evidence exists that both in mental health and other medical settings a valid communication has been associated with positive aspects for both patients, in terms of higher satisfaction in care processes, enhanced insight, improved adherence, and lower levels of litigation, and for caregivers, in terms of higher confidence, lower distress, and increased sense of wellbeing [6–9]. Moreover, good communication has been linked to better physical and mental health outcomes [10–13].

Establishing valid communication is not only a basic clinical need to be met but also a relevant ethical commitment. Application of the basic ethical principles of psychiatric practice is implemented largely by means of communication processes. Psychiatry shares with other medical specialties a set of common ethical principles. However, psychiatric ethics has been considered having a special status, due to the specific impact that mental suffering may produce on individual autonomy [14], the unique role of the therapeutic relationship, the vulnerability of people affected by mental disorders [15,16] and the increased risks of asymmetry in the mutual relationship between psychiatrists and their patients due to the power stemming from professional knowledge, institutional and legal role [4]. Moreover, particular ethical concerns derive from the stigmatization of mental illness [17].

The main reason for including families in a paper regarding ethical principles to be applied in communication is due to the awareness that, at least in Western countries, between 50 and 80% of patients affected by severe mental disorders are in close contact with their relatives [18]. This implies that family members are often involved in the role of “*informal caregivers*,” frequently being charged by a heavy emotional and practical burden [19,20]. According to the Madrid Declaration on Ethical Standards for Psychiatric Practice, the family should be consulted when a patient is “*gravely disabled, incapacitated and/or incompetent to exercise proper judgment because of a mental disorder*” [21]. Therefore, the family is an important interlocutor in treatment processes, even though this raises important ethical issues with regard to confidentiality [22–24].

In communication with individuals affected by mental disorders and with their families/caregivers a set of basic ethical principles should be accomplished (Table 1), namely: respect of patients’ autonomy, dignity, opinions, values, privacy and confidentiality, granting honesty and clarity, and tolerance of overt emotions’ expression [4,21,25–28]. Using an age and culture sensitive language, we should avoid time constraints which are among the worst enemy of communication and one of the most significant obstacles to “patient-centered psychiatry” [29], “shared decision making” [30], and to the implementation of psychotherapy [31], but several specific aspects should be specifically considered. The present paper aims to address ethical issues emerging in communication with patients and families in particular situations and contexts of psychiatric practice.

**Table 1.** Basic ethical principles in communication.

Respect patients’ autonomy, dignity, opinions, values, privacy, and confidentiality Grant honesty and clarity Tolerate overt emotions’ expression Use a culture and age sensitive language Avoid time constraints

## Communication about the Disorder

How to effectively and supportively communicate diagnosis is crucial for all medical specialties [32]. Doubts have been raised as to whether or not the diagnostic communication is handled adequately by mental health services [33,34]. Moreover, patients do not seem to be fully satisfied by their experience at the time of diagnosis [35], that is how the nature of their disorder is explained, which is its denomination, what are possible treatments and which are the possible outcomes. Diagnostic disclosure by clinicians, in particular, in the case of severe mental disorders, is generally considered an extremely challenging task. Indeed, a number of different factors have been reported to influence communication, including clinical conditions and level of insight of the patients, type of diagnosis and its level of uncertainty, the possibility of negative consequences for clients in terms of distress, social identity, demoralization, stigma, therapeutic relationship and treatment acceptation, and fears of stimulating negative emotional and behavioral reactions including suicidality [36]. As a consequence, clinicians are sometimes reluctant to communicate diagnosis in the case of severe mental disorders such as schizophrenia, often using more vague, substituting terms (e.g., psychosis), while the research conducted in this field, although limited, has prevalently reported the benefits to be gained from providing diagnostic information with no significant evidence of negative consequences [37]. Moreover, clinicians seem to underestimate the wish of service users to be informed about their condition [35,38], feeling that access to diagnostic information is their right and should be respected [37,38].

The right of patients to receive face-to-face information about diagnosis is widely acknowledged [37–44]. With regard to the therapeutic process, the Madrid Declaration [21] states that “*It is the duty of psychiatrists to provide the patient with all relevant information….*” Moreover, on discussing the issue of disclosing diagnosis in the context of Alzheimer’s Disease, the Declaration states that “*AD patient’s right to know is a well-established priority…*,” although it is acknowledged that *“at the same time, patients have the right also not to know if this is their wish. All must be given the opportunity to learn as much or as little as they want to know.”* Indeed, the *“right not to know”* has been applied from an ethical point of view to those disorders associated with stigma, irreversibility and lack of effective treatments [45]. Cultural issues may be relevant and should be taken into account in disclosing the diagnosis, given the potential significance of providing full disclosure in populations with considerably diverse sociocultural backgrounds [46]. In the light of collaborative practice, the clinician should approach the problem of communicating the diagnosis by first ascertaining what the patients know about their disorder, and what they want to know in terms of information, to provide a framework on which to guide discussion and identify the approach to be used [36]. Timing is an important issue to be taken into account, acknowledging that peoples’ desire to be informed may change over time, and that patients may not be well enough or may not be willing to receive information about their diagnosis in certain circumstances [39]. Thus, a flexible, stepped approach to disclosure of diagnosis may be the best means of meeting patient needs. In acute conditions, when the individual is severely distressed and insight may be lacking, together with the ability to collaborate and give consent, communication should, therefore, be delayed, allowing as much time as is needed to overcome the crisis and build up a trusting relationship [36]. Similarly, in the case of uncertain diagnosis, communication may be postponed, and patients should be informed of the need for additional time to evaluate their cases. In these circumstances, a provisional diagnosis may represent an ethically acceptable alternative [47]. In the communication of diagnosis *“the physician should give accurate and reliable information, using simple language*” [21]. Transparency of information, conveying hopeful information, utilizing collaborative approaches and being sensitive to stigma issues are considered of paramount importance in the diagnostic communication process [48].

The psychiatrist should provide information and discussion, respond empathically to patients’ emotions in relation to the news they receive, and respond to individual needs allowing further occasions to discuss the diagnosis and related issues [35,39]. Communication about the diagnosis should include an overall depiction of the nature of the disorder, its known determinants, course and known outcomes. Whenever possible, this basic information should be further enhanced through psychoeducation programs, which generally cover many other important aspects beyond diagnosis, such as illness management and treatments [49].

Confidentiality and privacy relating to diagnosis are not only an ethical obligation, but also an aspect of fundamental importance in the building up of a trusting relationship. Thus, communication with caregivers with regard to patients’ diagnosis and the breadth of information to be given should only be considered after discussing these issues with patients. Moreover, caregivers should only be informed once explicit consent has been obtained from patients. See Table 2 for a summary of ethical principles in communicating about diagnosis.

**Table 2.** Communication about diagnosis.

All patients should be informed about their diagnosis after having explored what they know and what they want to know about the illness Use a flexible, stepped approach to the disclosure of diagnosis In case of uncertainty, postpone the communication or provide a provisional diagnosis Discuss preliminarly with patients what kind and extent of communication about diagnosis should be given to caregivers

## Communication about the Disorder in Prodromal Stages

The “prodromal stage” is the time frame generally characterized by mild, nonspecific, or vague symptoms or signs which precedes the full-blown syndrome in a series of mental disorders. In recent decades, the introduction of specific semi-structured interviews, screening instruments, and diagnostic biomarkers have facilitated the early identification of people at risk of developing psychotic disorders such as schizophrenia, or neurocognitive disorders such as Alzheimer’s disease (AD). This possibility has given rise to a series of multifaceted ethical problems related to diagnostic communication, which are amplified by the paucity of information and studies reflecting patients’ and families’ perspectives with respect to prodromal stage. With regard to psychosis, the fact that only one third of those deemed to be “at risk” will develop psychosis over the ensuing 1–3 years [50] has raised the ethical question of if or when and how a psychiatrist should tell a patient that he/she is at an increased risk of developing psychosis. Major concerns have been expressed due to the fear that disclosure of this news may profoundly alarm patients and families, increasing the risk of stigma and labeling, with potentially negative consequences such as internalized stigma, identity engulfment and shame. These, in turn, may cause social retirement, curtailment of personal growth and achievements, increased stress, and enhanced risk of progression to psychosis [51–53]. It has been argued that respect of the “principle of autonomy,” which implies taking the risk of allowing the person to initiate an active intervention or monitor his/her state should be counterbalanced by the respect of the “nonmaleficence principle,” that is avoidance of harm. Thus, clinicians are faced with a significant ethical dilemma, considering that both full or partial disclosure and nondisclosure have benefits and caveats [54]. The tailoring of disclosure in line with factors and characteristics which are specific to each presenting case (the so-called “hybrid disclosure approach”) may be recommended as a possible solution to this dilemma [54]. According to this approach, parents or guardians (in the case of minors) and adults should be given full disclosure based upon a clear communication of what a “state at risk” means and what its prognostic implications are, which interventions may be implemented to address the problem and what lifestyle changes should be implemented to minimize the risks of psychosis. With regard to minors, information should be tailored to each individual, ranging from full disclosure to nondisclosure on the basis of a series of individual and contextual variables (e.g., age, cognitive capacity and level of insight, comorbidity, and suicidality). The other area of concern regards neurocognitive disorders. Patients who attend clinical centers for memory impairment may show early (prodromal) symptoms which are not yet sufficient to fulfill diagnostic criteria of dementia (e.g., mild cognitive impairment), while biomarkers provide evidence that dementia is highly likely to develop. In this case, should findings that indicate an uncertain risk of an alarming disease, be disclosed to the affected individuals? Significant ethical concerns have been raised in regard to prognostic uncertainty and lack of clinical utility associated with preclinical identification of AD [55]. Taking into account the absence of truly effective therapies and the possibly devastating impact of diagnosis for patients and families, the benefits of early diagnosis of AD have been strongly questioned [56]. Truthfulness and respect for autonomy may dictate the need for disclosure, being a pre-requisite for self-determination, also with regard to nonmedical decisions relating to the individual’s life choices [57]. However, the question of whether or not the diagnosis of possible AD is harmful to the patient when symptoms are still mild and the individual is not yet demented is currently undergoing debate [57].

In view of the complexity of this matter, from an ethical point of view, discussion, and negotiation relating to some aspects may be considered mandatory. In particular, the possibility of whether to receive or not the findings of medical examination should always be discussed with patients prior to evaluation, and the possibility of choosing whether to be informed or not left up to the patient, who may, of course, change his/her mind at any time. Moreover, patients’ understanding of the difference between common clinical practice and research should be carefully assessed [58]. Indeed, transparent communication relating to the research nature of examinations, the developing degree of scientific uncertainty, as well as the nature and amount of added value of the tests should be guaranteed, with a particular focus on respect for the self-determination and autonomy of the patient [57]. In the case of disclosure of mild cognitive impairment due to AD, the principle of “therapeutic privilege” may be applied if the physician feels obliged to forego full disclosure to safeguard the patient’s wellbeing, for example, situations in which full preservation of both the principles of autonomy and of beneficence may not be possible [59]. Following the finding of biomarker positivity, the physician should seek consent from the patient to allow a family member or other person to be informed of the outcome, based on the fact that active involvement of caregivers has been shown to enhance both the individual’s autonomy and beneficence [57]. See Table 3 for a summary of ethical principles in communicating about the disorder in prodromal stages.

**Table 3.** Communication about the disorder in prodromal stages.

Be aware that communication about the risk of developing psychosis may profoundly alarm patients’ and caregivers In case of prodromal states of psychosis, disclosure should be tailored in line with characteristics specific to each presenting case Disclosure should be accompanied by a clear communication about what the terms “prodromal” or “at risk state” really mean In case of suspected major neurocognitive disorders patients should be given the possibility of choosing whether to be informed or not the principle of “therapeutic privilege” may be applied if the physician feels obliged to forego full disclosure, in order to safeguard the patient’s wellbeing In case of biomarker positivity, the physician should seek consent from the patient to allow a family member or other person to be informed of the outcome

## Communication Relating to Therapeutic Options and Treatments (Informed Consent)

Informed consent is “the ongoing process that involves disclosing information important to the patient and/or the decision maker, ensuring the patient/decision-maker has the capacity to make treatment decisions and avoiding coercive influences” [60]. The principle of informed consent is based on the ethical standards of autonomy and self-determination. Information disclosure, voluntary choice, and decision-making capacity are considered the main components of informed consent [61]. In communication about any form of proposed treatments, whether pharmacological or nonpharmacological, the psychiatrist should illustrate the related risks and benefits, providing information as clearly as possible in line with the level of education and cultural background of the patients. They should be given the opportunity to ask for any additional information that they may require and the psychiatrist should ensure that the information provided has been properly understood. With regard to biological therapies implemented by means of psychopharmacological agents, Electroconvulsive Therapy (ECT), repetitive Transcranial Magnetic Stimulation (rTMS) or others, basic elements of knowledge should be given with regard to their effectiveness, how these treatments work, the period of time needed for them to be effective, how long their effects may last, what the expected adverse events are and how these may be overcome, and finally what the possible treatment alternatives are including no treatment at all. Illustrate possible advantages and disadvantages, in case no treatment choice. The same elements of knowledge should be given with regard to psychotherapies. In the case of an off-label use of treatments, the reasons, and scientific evidence underlying the proposed use should be illustrated. When dealing with the issue of side effects, the psychiatrist should be aware of the possibility, in some cases, of a “nocebo effect” of disclosure of side effects and the need to achieve a balance between the patients’ right to be informed with the ethical principle of nonmaleficence [62]. Psychiatrists should bear in mind that proper informed consent requires patients to be “competent,” meaning they are capable of understanding, remembering, and grasping information, and able to evaluate the impact of any decisions made and to communicate their decision. Accordingly, ethical concerns as to the validity of informed consent may arise in patients who are cognitively impaired [63], affected by acute mental disorder [64], or suffering from psychotic prodromal states [65]. Relatives may receive information unless they have been expressly prohibited from being notified of any relevant information relating clinical conditions of the patients and prescribed treatments. See Table 4 for a summary of ethical principles in communicating about treatments.

**Table 4.** Communication regarding treatments.

Provide information to patients about treatments, including their nature, related risks and benefits, time necessary for them to be effective, duration of effects, expected adverse events, and how these may be overcome Illustrate possible advantages and disadvantages in case if no treatment choice Be aware of the possibility, in some cases, of a “nocebo effect” of disclosure of side effects, balancing between right to be informed and principle of nonmaleficence Bear in mind that proper informed consent requires patients to be “competent” Relatives may receive information about treatments of their relative, unless they have been expressly prohibited

## Communication in the Case of Involuntary Treatments

Involuntary treatments include interventions legally established according to national laws and regulations, including psychiatric hospitalization, outpatient treatments, admission to residential facilities for therapeutic and rehabilitative purposes, and so on. Although involuntary treatments do not seem to be associated with a significant risk of negative outcomes, they may have a relevant impact on quality of life and treatment satisfaction [66].

Inherent ethical tensions between values relating to individual autonomy, provision of adequate patient care and community protection are intrinsically linked to enforced treatments, psychiatrists are therefore called upon to exercise these forms of involuntary intervention using the greatest possible sensitivity, finding a balance between these competing values, while respecting the informed consent process and patients’ decision-making capacity as much as possible [60]. Ethics dictate the need to inform patients with regard to any admissions to hospital and to describe diagnostic and therapeutic interventions that may be delivered [67]. In particular, patients should be given transparent information about the reasons for their involuntary treatment and the duration of the latter, together with the legal rights granted in the context of coercive treatment procedures. These ethical requirements apply to all forms and settings of involuntary treatment. Communication should be given in line with the clinical conditions of the subjects, and timely information provided throughout all steps of the procedure [67]. In particular, in case of aggressive or violent patients it will be appropriate to wait until the patient’s arousal has ceased before transmitting the necessary information. Should patients refuse to provide informed consent to treatments, providing of information related to patients’ condition to others should be avoided; however, every effort should be made to obtain informed consent to treatment with the aim of halting coercive treatment. Relatives may be involved in procedures relating to involuntary admission, and may provide useful information on the clinical conditions of the patient unless they have been expressly prohibited from being notified of any relevant information relating to the involuntary procedure, its presumable length and clinical conditions of the patients and prescribed treatments [67]. See Table 5 for a summary of ethical principles in communicating in case of involuntary treatments.

**Table 5.** Communication in case of involuntary treatments.

Give transparent information about the reasons for involuntary treatment, their duration and the legal rights granted in the context of coercive treatment procedures Describe diagnostic and therapeutic interventions that may be delivered Communicate according to the clinical conditions of the subjects, and provide timely information throughout all steps of the procedure In case of refusal of consent to treatments, do every effort to obtain informed consent with the aim of halting coercive treatment Relatives may be involved in procedures relating to involuntary admission unless they have been expressly prohibited from being notified of any relevant information

## Communication in the Case of “Dual Roles” (“Dual Agency,” “Overlapping Roles”)

In relation to their activities and different professional roles, psychiatrists *“may have competing obligations that affect interactions with patients”* [60]. These situations, in which respect of ethical principles of confidentiality and nonmaleficence may be questioned, are generally determined by forensic activities (i.e., participation as an expert in legal proceedings), or by other competency-related needs (e.g., assessment of fitness to work, suitability for specific work tasks and roles, to receive a disability pension, etc.). In these cases*, “it is the duty of a psychiatrist… to disclose to the person being assessed the nature of the triangular relationship and the absence of a doctor-patient relationship, besides the obligation to report a third party even if the findings are negative and potentially damaging the interests of the person under assessment*” [20]. Although the treating psychiatrist should prioritize patient interest, they should *“reconcile these interests against other competing commitments and obligations*” [60]. Even when the psychiatrist is acting as a consultant, the above-mentioned obligations should be pointed out to their clients. Psychiatrists may breach the principle of confidentiality only to the extent that disclosure is required for forensic functions, and to comply with the ethical commitment of personal respect. Otherwise they should avoid any disclosure of information provided by the patient beyond the confines of the process, for example during conversations with the media [68]. Should psychiatrists be called to serve as an expert for a patient under their care, prior to accepting the task, they should discuss this possibility with their patient and remind them of their ethical and legal obligation to *“tell the truth”* [68], and of the potential breach of confidentiality highlighting the risk that their testimony could result in unintended outcomes and adverse effects, including breakdown of the therapeutic relationship, discussing the lack of scientific precision in the legal process, and the possibility of negative decisions by the court [60]. This candid discussion should allow the patient to weigh up the risks and benefits involved if the treating psychiatrist decides to testify or not [60]. See Table 6 for a summary of ethical principles in communicating in case of “dual roles.”

**Table 6.** Communication in case of “dual roles.”

In case “dual roles” disclose to the person to be assessed the absence of a doctor-patient relationship and the obligation to report a third party Acting a consultant, inform the patient that you may breach the principle of confidentiality only to the extent that disclosure is required for forensic functions Avoid any disclosure of information provided by the patient beyond the confines of the process, including conversations with the media Serving as an expert for patients under your care, remind them of your ethical and legal obligation to “tell the truth”

## Communication in Genetic Counseling

Following recent reports demonstrating how the risk of mental disorders such as schizophrenia, bipolar disorders, and autism spectrum disorders may be significantly increased in individuals who test positive for specific genetic markers, psychiatric counseling has shifted from the simple estimation of family history-based risk to estimates deriving from specific and, at times, sophisticated tests [69]. As a consequence, the ethical concerns are now focused on abortion and selection of embryos for implantation on the basis of the probability of a psychiatric disorder, the rights of family members to receive genetic information regarding other members, risk of stigmatization for the member who is a carrier, stigma deriving from community, and ethnicity-based genetic information, the role of genetic test in marital choice and of populations’ genetic screening and prevention programs [69].

Psychiatrists should take particular care in communicating with patients and families over genetic risk issues, providing updated information of the current state of the art in the field, and making it clear that current genetic knowledge is still incomplete, as being a developing issue, the future findings may alter the current notions [20]. When genetic testing is requested, patients and families should be referred to reliable specialist facilities [20]. Psychiatrists should bear in mind that genetic information will not affect the interested individual alone, as disclosure of results may generate negative and disruptive effects, also for other family members [20]. Psychiatrists should explicitly discuss with the patient the opportunity of sharing genetic information with family members in order to obtain explicit consent to disclose information and receive an indication of the extent to which the patient wishes them to be involved in communication. As a routine, counselors should consider the ethical implications of genetic disclosure and the complexity of psychological consequences and be prepared to offer psychotherapeutic support as part of the consultation process, in order to address interpersonal issues and narcissistic injuries which may arise from genetic results [69]. Genetic counseling regarding family planning and abortion should comprise all information needed to assist patients in reaching a decision; in these cases, psychiatrists should be particularly respectful of patients’ values and decisions [20]. See Table 7 for a summary of ethical principles in communicating in case if genetic counseling.

**Table 7.** Communication in genetic counseling.

Take particular care in communicating with patients and families over genetic risk issues, providing updated information of the current state of the art in the field Make it clear that current genetic knowledge is still incomplete, as being a developing issue Be aware that disclosure of results may generate negative and disruptive effects bot for patients and other family members Discuss with the patient the opportunity of sharing genetic information with family members and obtain explicit consent to disclose information Counselors should consider the ethical implications of genetic disclosure and the complexity of psychological consequences and be prepared to offer psychotherapeutic support as part of the consultation process In case of genetic counseling for family planning and abortions, be respectful of patients’ values and decisions

## Communication in End-of Life Conditions

Psychiatrists collaborating with palliative care teams have increased significantly in recent decades, frequently practicing under the label of palliative care psychiatry [70]. Psychiatrists are often involved in the evaluation and treatment of end-of-life patients, playing a crucial role due to their experience in dealing with sensitive and difficult discussions with patients [60]. “*Palliative psychiatry*” provides support to patients, including those affected by severe mental disorders, in coping with and accepting distress, helping them to live as actively as possible until death, enhancing quality of life, and supporting families in coping with end-of-life patients [71]. Successful formulation and implementation of end-of-life care rely largely on ongoing communication with the patient [72]. Talking overtly about death and dying, assuming the perspective of considering death not as a failure of medicine but rather as a natural event, ensuring that the patient will not be abandoned and that every effort will be made to relieve pain and suffering, should be core aspects of communication with patients in these circumstances [73]. Psychiatrists are expected “*to be truthful with patients about their diagnoses and prognosis*” and “*must have the requisite compassion and skill to thoughtfully and sensitively foster dialogue with patients who are seriously ill and suffering from a terminal illness*” [60]. See Table 8 for a summary of ethical principles in communicating in end of life conditions.

**Table 8.** Communication in end-of life conditions.

Provides support to patients, including those affected by severe mental disorders, in coping with distress Help them to live as actively as possible until death, enhancing quality of life, and support families in coping with end-of-life patients Talk overtly about death and dying ensuring that the patient will not be abandoned and that every effort will be made to relieve pain and suffering Be truthful with patients about their diagnoses and prognosis

## Data Availability

No publicly available data have been used to support the present paper.
